# Deep Learning for Single-Shot Structured Light Profilometry: A Comprehensive Dataset and Performance Analysis

**DOI:** 10.3390/jimaging10080179

**Published:** 2024-07-24

**Authors:** Rhys G. Evans, Ester Devlieghere, Robrecht Keijzer, Joris J. J. Dirckx, Sam Van der Jeught

**Affiliations:** 1 Industrial Vision Lab (InViLab), Faculty of Applied Engineering, Campus Groenenborger, University of Antwerp, Groenenborgerlaan 179, 2020 Antwerp, Belgium; sam.vanderjeught@uantwerpen.be; 2 Laboratory of Biomedical Physics (BIMEF), University of Antwerp, Groenenborgerlaan 171, 2020 Antwerp, Belgium

**Keywords:** 3D imaging and sensing, machine learning, profilometry, structured light, dataset

## Abstract

In 3D optical metrology, single-shot deep learning-based structured light profilometry (SS-DL-SLP) has gained attention because of its measurement speed, simplicity of optical setup, and robustness to noise and motion artefacts. However, gathering a sufficiently large training dataset for these techniques remains challenging because of practical limitations. This paper presents a comprehensive DL-SLP dataset of over 10,000 physical data couples. The dataset was constructed by 3D-printing a calibration target featuring randomly varying surface profiles and storing the height profiles and the corresponding deformed fringe patterns. Our dataset aims to serve as a benchmark for evaluating and comparing different models and network architectures in DL-SLP. We performed an analysis of several established neural networks, demonstrating high accuracy in obtaining full-field height information from previously unseen fringe patterns. In addition, the network was validated on unique objects to test the overall robustness of the trained model. To facilitate further research and promote reproducibility, all code and the dataset are made publicly available. This dataset will enable researchers to explore, develop, and benchmark novel DL-based approaches for SS-DL-SLP.

## 1. Introduction

SS-DL-SLP was first introduced in 2019 [1,2,3]. Initially, researchers utilised simulated height maps to demonstrate that a full-field 3D height map could be reconstructed from a single deformed fringe pattern through a trained neural network. A random surface map generator produced an extensive training dataset of simulated height maps with corresponding deformed fringe patterns. A neural network was trained on the resulting data couples to approximate a mapping function that transforms a 2D array of grayscale values (representing the deformed fringe pattern) to a continuous 3D height distribution. The advantage of deep learning in this context is that the network can extract such a height map from only a single deformed fringe pattern, in contrast to standard phase-shifting profilometry techniques that require three or more distinct patterns to reconstruct the 3D surface map analytically. In addition, the network can be trained to learn intermediate data processing steps, such as noise reduction, background masking, and phase unwrapping, as part of its mapping function.

Many research groups have since been actively working on implementing the technique in real optical profilometry setups and improving the performance of the employed neural network. Such improvements can be seen in the model architectures ranging from the traditional CNNs [4] and autoencoders (U-NETs) [5] to more recent architectures, such as split autoencoders (Y-Net, DY-Net++) [6,7,8,9,10] and generative adversarial networks (GANs) [11]. Other improvements can be achieved by optimising the loss function [12,13,14,15] or the employed projection pattern [16,17,18].

Although progress has been made in optimising various network features, the absence of standardised datasets for training and evaluation purposes presents a significant challenge in gauging the relative impact of these efforts. Each research group has mostly depended on its distinct datasets of 3D scenes, which often exhibit variations in scene complexity, illumination conditions, surface materials, and noise levels. As a result, the lack of benchmark datasets poses several obstacles:

- Without a common ground for comparison, it becomes challenging to determine whether accuracy, robustness, or efficiency improvements result from algorithmic advancements or dataset-specific idiosyncrasies;
- The lack of a standardised dataset impedes the reproducibility and replicability of experiments. Other researchers aiming to validate or build upon existing methods may struggle to obtain similar or consistent results because of disparities in dataset characteristics. This hinders the field’s progress and slows the adoption of novel techniques;
- Different datasets often demand tailored evaluation metrics for their specific challenges. Without a common dataset, there is no consensus on which evaluation metrics are most appropriate for assessing the performance of DL-SLP methods. Consequently, comparing performance across studies becomes convoluted and lacks a clear reference point;
- Real-world applications of structured light profilometry require robustness to diverse scenarios and environments. Research groups’ reliance on distinct datasets may result in models that perform well on specific datasets but struggle when confronted with different scenes.

These obstacles limit the developed methods’ practical utility and inhibit their adoption in real-world applications. To illustrate the disparity between datasets used in SS-DL-SLP, Table 1 lists the respective object types, number of images, and recording methods used in several recent studies. All datasets were manually recorded and are physical recordings. This means that personal knowledge and bias could have been present during the dataset creation, consciously or subconsciously, to achieve better results.

We excluded computer graphic datasets as these have only recently shown applicability in real-life situations, with again specifically chosen data examples used for training the network [5]. In the following, we present a dataset of height maps with corresponding deformed fringe patterns obtained from real images and extracted from a physical calibration target. Statistical dataset analysis is included to ensure an even distribution in surface modulation between data samples.

## 2. Materials and Methods

### 2.1. Creation of the Dataset

**Figure 1:**
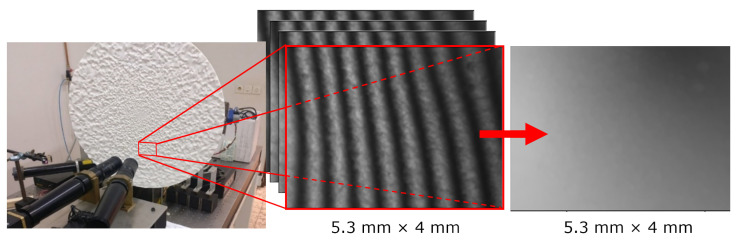
Automated data gathering setup featuring a 3D-printed calibration target containing randomly fluctuating geometry (**left**). During a single measurement of a subsection of the calibration wheel, four phase-shifted fringe patterns are projected, and the deformed patterns are recorded (**middle**). These are combined to form a 3D height profile (**right**).

To generate a dataset for DL-SLP, a calibration target featuring random surface variations was designed and 3D-printed (Figure 1). The surface geometry of the target contains peaks and troughs whose gradients increase toward the centre of the target. Both smooth Gaussian peaks and well-defined edges are included in the surface profile. The developed surface profile and dataset is called the Gaussian Depth Disc (GDD). We generated our dataset autonomously, ensuring that all images were unique. We conducted processing and statistical analysis to verify this uniqueness and to identify any outliers or similarities among the images. The randomised local geometry variations are critical to enhancing the practical utility of DL-SLP in real-world contexts. The GDD dataset closely emulates the inherent variability of objects encountered in realistic environments by encompassing a diverse range of shapes and surface profiles.

Materialise, Inc., employed a selective laser sintering (SLS) process to 3D-print the circular target, which had a radius of 540 mm and spanned a maximum depth of 5 mm. To scan the target’s surface, we set up a standard 4-step phase shift measuring system. This setup integrated a digital light projector (Texas Instruments Lightcrafter evaluation module) and a digital camera (IGV-B0620M, capturing 210 frames per second with images of 640 × 480 pixels in 12-bit grayscale). We employed telecentric lenses to minimise geometric distortions. Each captured image covered an area of 5.3 × 4 mm. To ensure synchronisation, we created custom electronics that linked the trigger output of the TI module logic board to the high-speed camera. The camera’s exposure time matched the interval between consecutive trigger pulses, resulting in 8.3 ms per image capture. Previous research established the depth resolution for this arrangement to be <50 µm RMS per pixel [23]. After four successive recordings, we combined the projected fringe patterns deformed by the surface geometry to form a (wrapped) phase map. After phase unwrapping, the phase map and its respective deformed fringe pattern were stored in a central PC. Next, the calibration target was rotated automatically using a stepper motor over a predetermined angle, and the cycle was repeated. When a full 360° rotation of the calibration target was complete, the target was shifted vertically to measure a different radial band. Following this procedure and allowing ample time for the calibration target to rotate and stabilize in each new position, approximately 1000 phase maps could be recorded per hour this way.

### 2.2. Data Analysis

After gathering a full dataset of 18,829 data couples, analysis was conducted to verify that it contained a representative distribution of surface modulations. Ideally, the dataset would include a somewhat evenly distributed mix of flat (low-gradient) and jagged (high-gradient) surface maps. To gauge this, several statistical properties of each height map were evaluated, including the height map’s depth range and the standard deviation. The range indicates the difference in height between the highest and lowest points of the surface, while the standard deviation measures the amount of dispersion around the centroid of the height map. It should be noted that these two characteristics alone do not unambiguously reflect the amount of surface modulation in every aspect. Indeed, a low standard deviation value can mean that the surface map is either noisy with a small range or noise-free with an arbitrarily large amount of surface modulation. Therefore, having a third measure that indicates the overall modulation of the surface height map is helpful. A measure based on the local gradient can provide a high value for these types of surfaces, irrespective of the total height difference. The Root Mean Square Gradient (RMSG) [24] function generates a low value for flat surfaces with little height modulation and higher values for jagged surfaces with large modulations. Formally, the RMSG can be written as [24]

(1) $$RMSG\;=\;\sqrt{\frac{1}{\left(n_{x}-1\right)n_{y}}\sum_{i=1}^{n_{x}-1}\sum_{i=1}^{n_{y}}{\left(\frac{\partial z}{\partial x}\right)}^{2}\;+\;\frac{1}{n_{x}\left(n_{y}-1\right)}\sum_{i=1}^{n_{x}}\sum_{i=1}^{n_{y}-1}{\left(\frac{\partial z}{\partial y}\right)}^{2}}$$

where

(2) $$\left(\frac{\partial z}{\partial x}\right)=z\left(x_{i+1},y_{j}\right)-z\left(x_{i},y_{j}\right)$$

and

(3) $$\left(\frac{\partial z}{\partial y}\right)=z\left(x_{i},y_{j+1}\right)-z\left(x_{i},y_{j}\right)$$

If we consider that $z(x_{i},y_{j})$ represents the height of the surface map at row *i* and column *j* and $n_{x}$ is the number of rows while $n_{y}$ is the number of columns of the given surface, then the derivative with respect to *x* can be calculated up to row $n_{x}-1$, and the derivative with respect to *y* can be calculated up to column $n_{y}-1$.

**Figure 2:**
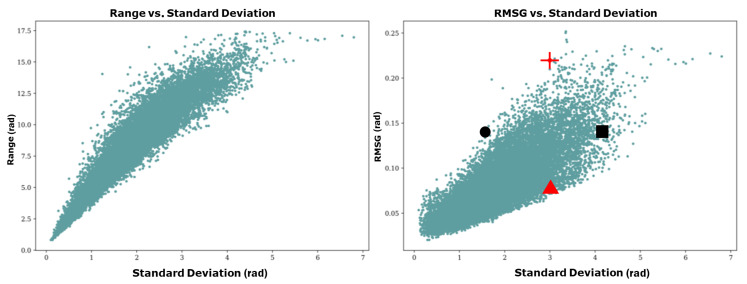
Scatter plots illustrate the spread of each pair of data points in the dataset. The plots show the range, which is the distance from the lowest pixel value to the highest pixel value in each image, plotted against the standard deviation, which is calculated with reference to the mean value of the image (on the **left**). The scatter plot (on the **right**) shows RMSG (see Formula (1)) plotted against the standard deviation. Two black samples (the circle and square) and two red samples (the triangle and cross) are selected to compare the surfaces, see Figure 3 for further information.

**Figure 3:**
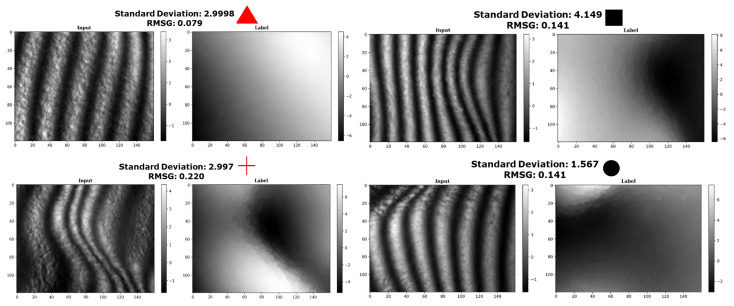
A mosaic containing comparable samples, of which two red data samples (namely the red triangle and red cross) from Figure 2 with similar standard deviations but with different RMSGs and two black data samples (namely the black square and black circle) from Figure 2 with similar RMSG but with different standard deviations.

Figure 2 plots the range versus standard deviation and the RMSG versus standard deviation of each sample in the dataset. Upon observation of the scatter plot, it becomes apparent that there is a stronger correlation between range and standard deviation than there is between RMSG and standard deviation, justifying the introduction of the RMSG as a more representative classifier of the dataset. To illustrate this further, four samples are highlighted in the plot: two samples with similar standard deviations but differing RMSGs (red triangle and red cross) and two samples with similar RMSGs but differing standard deviations (black circle and black square). These samples are illustrated in Figure 3.

**Figure 4:**
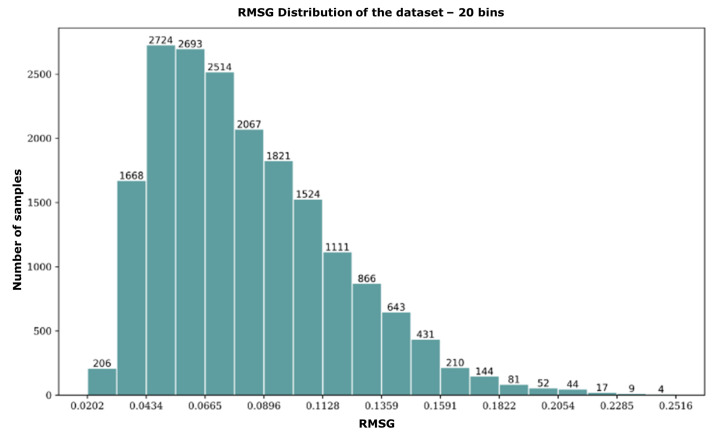
Histogram of the dataset showing 20 discrete bins based on their respective RMSG values. The number of images within each bin is denoted on top of each bar.

The height maps of the red triangle data couple and the red cross data couple have nearly identical standard deviations. However, upon inspection, it is clear that they exhibit a very different surface modulation. The red triangle sample has a smooth, gradual surface modulation, while the red cross sample exhibits strong modulations in the centre of the map. When comparing their respective RMSG values, however, they do differ significantly. We repeat this process along the RMSG axis by comparing the black square and black circle samples with similar RMSG values but different standard deviations. Despite having different surface shapes, it is still reasonable to classify them in the same “surface modulation category”, which strengthens the argument for RMSG as the superior statistical descriptor of surface modulation. In the following, we categorise all data samples into 20 discrete bins based on their respective RMSG values. A histogram of this distribution is included in Figure 4. A critical aspect of the RMSG method is that it compares neighbouring pixels, which means that image noise can cause the RMSG value to increase beyond the actual data. However, we can use this feature to our advantage when manipulating the data, as is demonstrated in the next section.

### 2.3. Data Manipulation

After binning the samples in our dataset based on RMSG in a histogram (Figure 4), it becomes apparent that the data representation is non-uniform. The histogram shows that there are more samples with low RMSG values than those with high RMSG values in the dataset. This indicates an over-representation of flat surfaces with relatively low surface modulation. Such results can be explained by the design of the calibration wheel, where the outer radius of the wheel contains larger bands of relatively flat surfaces. It is preferable to have a more even distribution of surface modulations to better reflect reality when using the dataset to train a neural network. To improve the dataset in this regard, the samples with under-represented RMSG values are duplicated artificially. This involves mirroring the fringe patterns (and their corresponding height maps) in a particular bin along the central horizontal axis. By doing this, the number of samples within that bin is effectively doubled. It is important to note that the samples are mirrored along the horizontal axis since fringe patterns are projected vertically in our measurement setup.

### 2.4. Asymmetry Index

When including a mirrored image of the sample in the dataset, the original and the mirrored sample must be sufficiently different to be considered a new sample. Some surfaces that are relatively symmetrical around the horizontal axis are not different enough after mirroring to allow for duplication. To express the similarity between the upper and lower half of samples quantitatively, the per-pixel RMS difference between both halves is calculated:

(4) $$RMSE\left(upper/lower\right)\;=\;\sqrt{\frac{1}{\frac{n_{x}}{2}\times n_{y}}\sum_{i=1}^{\frac{n_{x}}{2}}\sum_{i=1}^{n_{y}}{\left[z\left(x_{i+1},y_{j}\right)-z\left(x_{n_{x}+1},y_{j}\right)\right]}^{2}}$$

where $z(x_{i},y_{j})$ represents the height at row *i* and column *j* and, respectively, $n_{x}$ and $n_{y}$ indicating the number of rows and columns. The more asymmetric the upper and lower halves of a data sample, the higher the RMSE (upper/lower). In the case of a perfectly symmetrical surface, the upper half of the surface is equal to the mirrored image of the lower half. This results in an RMSE of zero. It is important to note that the RMSE value also depends on the overall spread of the data. If the data have a large standard deviation, the RMSE value is automatically higher, even if the asymmetry between the upper and lower parts is not significant. To arrive at the ‘Asymmetry Index’, we divide the RMSE by the standard deviation of the sample:

(5) $$\mathrm{assymetry}\;\mathrm{index}=\frac{RMSE(upper/lower)}{\mathrm{Standard}\mathrm{Deviation}}$$

**Figure 5:**
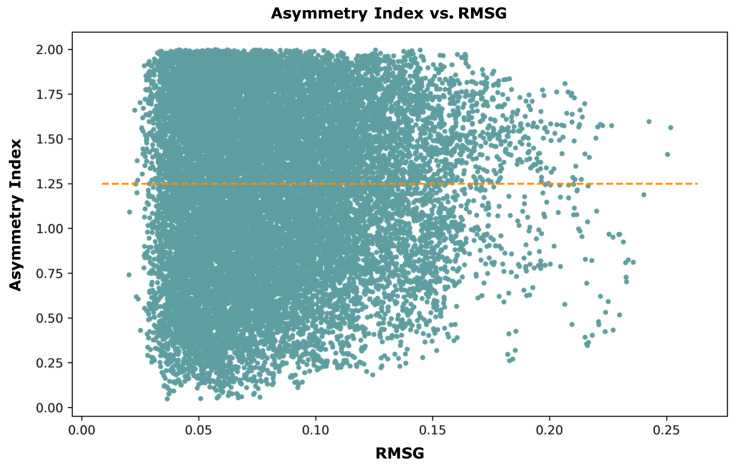
Scatter plot showcasing the asymmetric properties of each dataset couple and whether it has a value greater than or equal to 1.25.

Figure 5 displays our dataset’s distribution of RMSG and Asymmetry Index. According to the graph, the index value peaks at 2. This result is a consequence of the ratio between the standard deviation of the entire surface and our RMSE (upper/lower). We set the threshold for data augmentation to occur at an asymmetry index of 1.25. This means that surfaces with an index higher than 1.25 are asymmetric enough to include the mirrored image as a separate sample. This adds around 50% of samples to the corresponding bin. In Figure 5, the data points above the orange dotted line meet this criterion.

**Figure 6:**
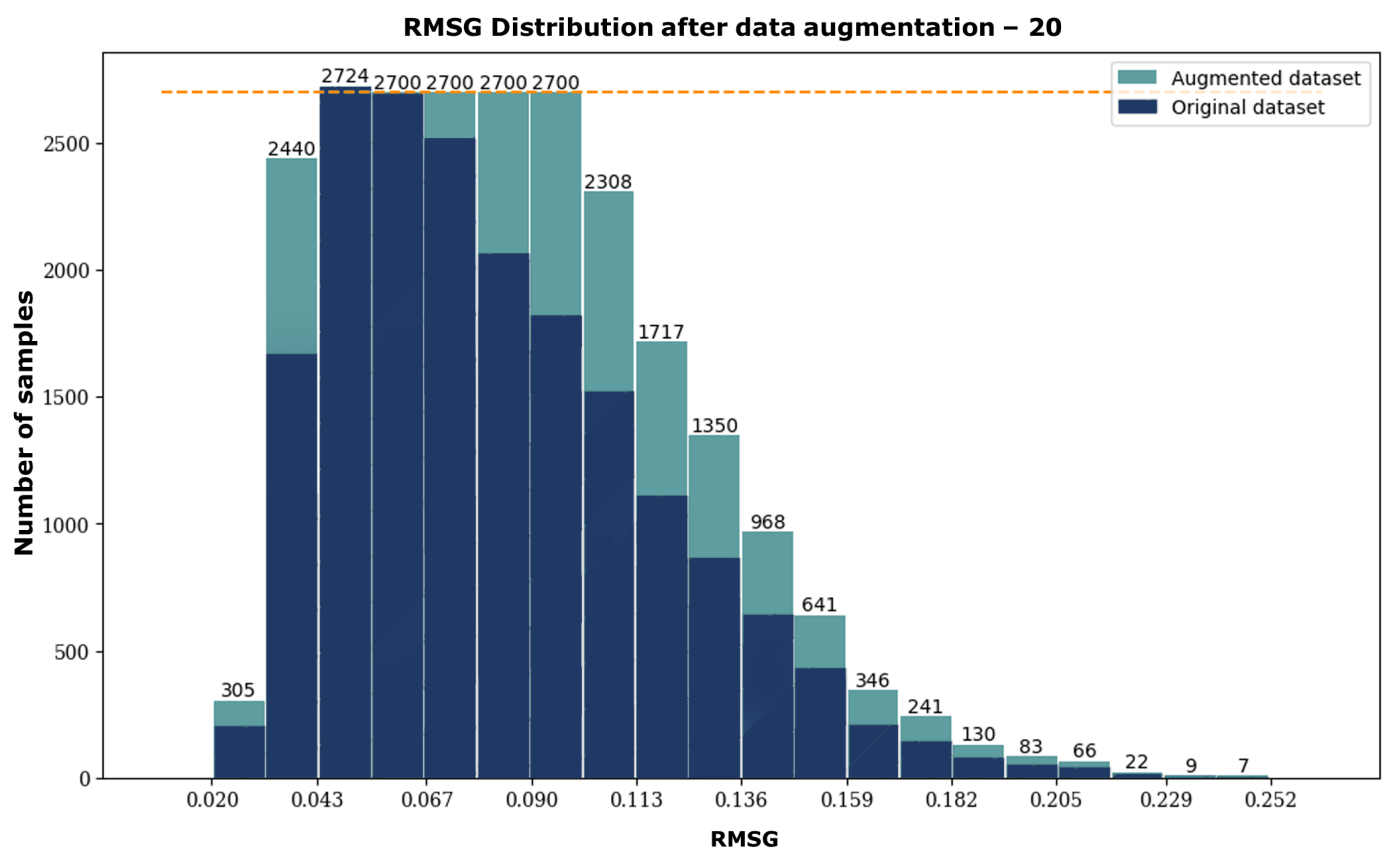
Histogram of the augmented dataset showing 20 discrete bins based on their respective RMSG values. The number of images within each bin is denoted on top of each bar. The original histogram is included in dark blue, and the samples added through data augmentation are included in teal.

Additionally, to prevent an imbalance of data, we set a hard limit of 2700 samples per bin, corresponding to the maximum amount of samples in a single bin in the original histogram. This data augmentation process leads to a more uniform data distribution, as can be seen in Figure 6.

The largest bin containing 2724 samples remained unchanged. Four other bins were enlarged up to the threshold of 2700 samples. All other bins below this threshold were maximally doubled. When visually comparing the two histograms, it becomes apparent that more samples were added to the middle bins than to the extreme bins. For instance, the second-largest RMSG bin remained unchanged, possibly because the samples were less asymmetric. The original dataset comprised 18,829 samples and was expanded to 24,157 after data augmentation. Consequently, a total of 28% new samples were added to the dataset. The augmented dataset is publicly available, including Python scripts that support training, editable data distribution, and validation. In addition, the original Matlab script to design the calibration target is included here. We included the original file format in the publicly available dataset, which is 480 × 640. Please find the DOI reference below to explore the dataset and access the files on Zenodo: https://zenodo.org/doi/10.5281/zenodo.10404433 accessed on 7 July 2024.

## 3. Results

Several established deep learning models were trained on the dataset to evaluate how well the surface maps generalise toward unseen data. The models were then used to infer the surface map of previously unseen scenes based on their respective deformed fringe patterns. The training termination criteria were standardised to ensure fairness among all models, resulting in a consistent training period of 200 epochs for each model. All models employed Adam optimisation with a dynamic learning rate starting at $1\times 10^{-3}$. The models use a 5 × 5 kernel size with L2 + regularisation loss. Note that the models generate radial phase values rather than absolute distance values. Specific hyperparameters and predictive accuracy are presented in Table 2. Before training, we normalised the data range to [0, 1 rad] and rescaled it to its original range after training. Throughout the analysis of the height prediction performance, we use radians as the unit of choice, and we use the relative RMS error to evaluate prediction performance. Given the scalability of structured light profilometry in general, this allows for more general conclusions to be drawn in the function of the employed field of view.

**Figure 7:**
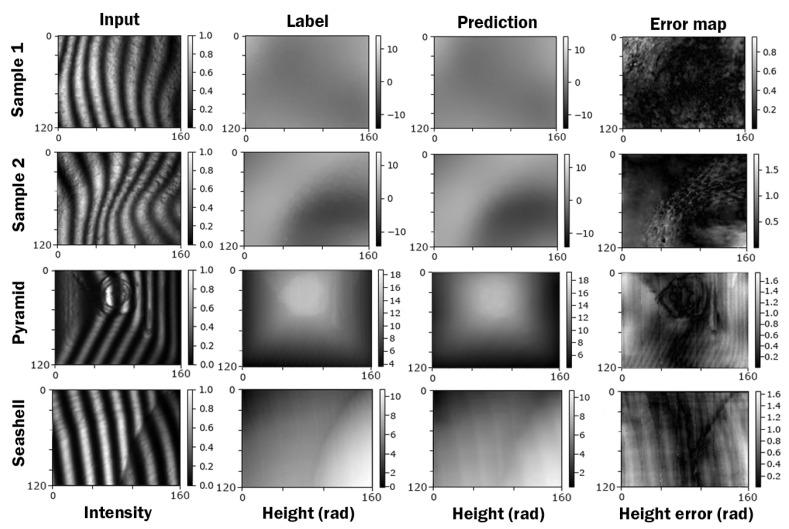
Mosaic plot of four samples, each with input fringe pattern, ground truth label, model prediction, and error map. The first two samples were taken from the GDD dataset, and the pyramid and seashell data captures are physical samples outside of the GDD dataset.

After training, Dense-Net achieved a validation loss of 0.01758, which can be interpreted as a prediction error of well below 2% of the height range. A selection of samples is included in Figure 7. The selected four samples are Samples 1 and 2, which are images taken from the training dataset, and a pyramid and a seashell, which are objects we captured and used to validate the performance of our network outside the direct scope based on what the networks were trained. It can be seen that the network can reconstruct the 3D geometry of previously unseen scenes very well. Although the network prediction error is generally low (<2%), it can be seen in the error maps that some high-frequency information is lost. Nevertheless, any further optimisation of the networks to further improve the high-frequency prediction quality is outside the scope of this work.

**Figure 8:**
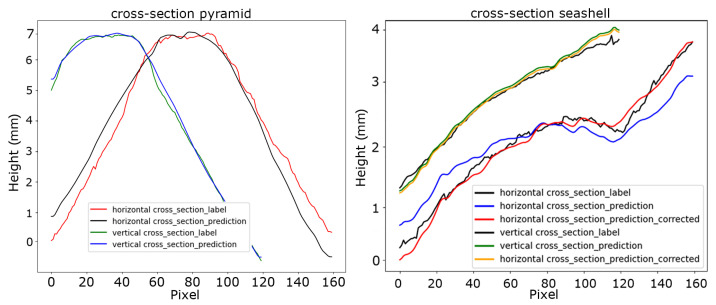
Horizontal and vertical cross-sections of the pyramid and seashell measurements when reconstructed using standard phase-shifting profilometry (red and green) and the corresponding network prediction (blue and black). Including skew-corrected predictions (red and yellow) in the Seashell cross-section.

Figure 8 shows the cross-sections through the peak of the pyramid (left) and the central cross-sections of the seashell (right). The blue and green plots in the pyramid graph correspond to the vertical cross-sections of the prediction and the ground truth, respectively. Along this dimension, a very close match between the ground truth and the prediction can be observed. The black and red graphs correspond to the horizontal cross-sections of the prediction and the ground truth, respectively. Here, the error between prediction and ground truth is considerably larger. In this regard, it should be mentioned that subtraction of the reference plane, as is custom in standard phase shifting profilometry, now happens directly as part of the network’s mapping function. Upon analyzing the horizontal cross-section of the prediction, we observe that the surface appears to be slightly skewed in comparison to the ground-truth surface.

This may indicate that the orientation of the object was different from that of the calibration wheel during the collection of the training dataset. It can be noted that the skew has a much less profound effect on the vertical cross-section of the predicted height map, which is presumably due to the orientation of the projected fringe patterns. The same effect was noticed in the measurement of the seashell. A cross-section along the horizontal and vertical axes of the network prediction is included in Figure 8 on the right. Notably, for this instance, we applied a vertical skew correction of 0.54° to enhance the alignment between the predicted and ground-truth surface. The skew correction significantly reduced the prediction error from 2.16% to 0.75%.

### Sine Wave Robustness

**Figure 9:**
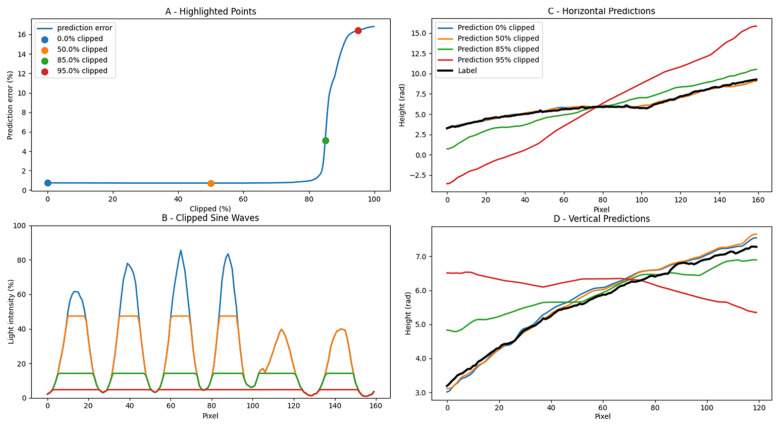
Degradation of prediction accuracy in function of reduced sine wave contrast.

We analysed the importance of capturing a perfectly modulated sine waveform in the data-capturing process. To accomplish this, we digitally manipulated the recorded images to progressively reduce the contrast of the sine waves and essentially converge them into block waves before feeding them to the network. The results are shown in Figure 9. Four key points on the resulting RMSE graph are selected to monitor the decline in prediction quality. The corresponding cross-sections of the seashell measurement are included in Figure 9B when a reduction of 0%, 50%, 70%, and 85% of the maximum intensity is applied. In Figure 9A, it can be seen that the network performs well up until a contrast reduction of 70% of the sine wave amplitude is applied. Beyond this threshold, a discernible decline in quality becomes apparent. At an 85% loss of the sine wave, a pronounced deterioration in quality is observed, and the 3D shape can no longer be accurately reconstructed. To assess prediction quality, vertical and horizontal cross-sections were conducted to observe the degradation of quality, as illustrated in Figure 9C,D. These cross-sections demonstrate that as the sine wave deteriorates, the prediction quality worsens. Notably, the model exhibits robustness in handling deviations in the projection quality of input images. It should also be noted that further complexities, such as surface roughness changes and opaqueness differences, are not present in the dataset and will be included in future works to further improve upon the currently available dataset.

## 4. Conclusions

A new Gaussian Depth Disc (GDD) dataset containing height maps and corresponding deformed fringe patterns is presented. It was assembled using a physical, automated calibration target. Statistical analysis was performed to maximise the surface modulation distribution within the dataset, and rudimentary robustness analysis was performed. It was demonstrated that the presented dataset can be used to accurately predict the height map of previously unseen targets within an RMS error range of <2%. The dataset is made freely available to the community to serve as a benchmark for future single-shot, deep learning-based structured light profilometry.

## Figures and Tables

**Table 1:** Datasets for structured light profilometry found in literature.

Dataset Name	Objects Type(s)	Dataset Size (# Images)	Generation Process
FP672 Nguyen et al. [2]	Four Disney dolls (together and separately)	672 images	Manual
FP1000 Li et al. [19]	Bust/doll/small items	1000 images	Manual
FP147 Song et al. [13]	Busts	147 images	Manual
Unnamed Bai et al. [20]	Dental casts (eight casts)	2755 images	Manual
Unnamed Qiao et al. [21]	Phone case glass (Samsung, iPhone, glass plates)	1200 images	Manual
Unnamed Nguyen at al. [22]	Clay sculptures	1500 images	Manual
GDD	Random Gaussian Disc	24,157 images	Automatic

**Table 2:** Hyperparameters and performance of each model when trained on the proposed dataset. The same training and validation fractions were used between different models.

	Channels	Number of Parameters	Loss Train	Loss Validation
CNN [2]	16, 16, 32, 64, 128	448k	0.05071	0.05108
Dense-Net [2]	16, 32, 64, 128, 64, 32, 16	1213k	0.01690	0.01758
U-Net [19]	16, 32, 64, 128, 64, 32, 16	454k	0.05000	0.05033

## Data Availability

All data, necessary code and files can be found in the Zenodo repository through the following link: https://zenodo.org/doi/10.5281/zenodo.10404433 accessed on 7 July 2024.
